# Xerostomia in patients with sleep apnea-hypopnea syndrome: A prospective case-control study

**DOI:** 10.4317/jced.56593

**Published:** 2020-08-01

**Authors:** José Pico-Orozco, Marina Carrasco-Llatas, Francisco-Javier Silvestre, Javier Silvestre-Rangil

**Affiliations:** 1 DDS, private practice; 2 MD, PhD. ENT Department. University Dr. Peset hospital, Valencia, Spain; 3 MD DDS PhD. Department of Stomatology, University Dr. Peset hospital, Valencia. Special Care in Dentistry, University of Valencia, Spain; 4 DDS, PhD. Special Care Dentistry, Department of Stomatology, University of Valencia, Spain

## Abstract

**Background:**

To describe the characteristics of xerostomia (dry mouth) in the population with sleep apnea-hypopnea syndrome (SAHS) and contrast its prevalence versus that found in healthy subjects, and to compare the frequency of xerostomia in SAHS patients with and without continuous positive airway pressure (CPAP) treatment.

**Material and Methods:**

A prospective comparative study was made between adults recently diagnosed with SAHS in a public hospital (n=60) and healthy individuals (n=54). The presence of xerostomia was assessed on waking up and during the day, using a frequency scale from 0 (“never”) to 3 (“always”).

**Results:**

The prevalence of xerostomia on waking up in the SAHS group was 45%, versus 20.4% among the controls. During the rest of the day the prevalence of the symptom decreased in both the SAHS group (21.7%) and among the controls (9.3%). Multiple binary logistic regression analysis found body mass index (BMI) to be correlated to the association SAHS-xerostomia on waking up (*p*=0.007). Patients with moderate SAHS had a greater frequency of xerostomia than those with mild SAHS (*p*=0.022). The frequency of xerostomia on waking up was significantly greater in patients using CPAP than in those without such treatment (57.1% versus 16.7%, respectively) (*p*=0.008).

**Conclusions:**

The frequency of xerostomia was greater in patients with SAHS, particularly on waking up and in those receiving CPAP. The symptom was more prevalent in individuals with moderate to severe SAHS than in those with mild SAHS, and was significantly influenced by BMI.

** Key words:**Sleep apnea, xerostomia, dry mouth, CPAP, side effects, body mass index, obesity.

## Introduction

Sleep apnea-hypopnea syndrome (SAHS) is a major public health problem in view of its high prevalence and medical, social and economic impact (1). Total or partial interruption of breathing activity repeatedly during sleep causes hypoxia and sleep fragmentation, and is associated to cardiovascular, metabolic and neurocognitive disorders (2). The condition affects 3-17% of the adult population (3) and is more common in males, obese individuals and menopausal women (4). The most frequent clinical manifestations are snoring and excessive daytime sleepiness. The gold standard for diagnosing and assessing the severity of SAHS is polysomnography (PSG) (5), based on the recorded apnea-hypopnea index per hour of sleep (AHI/h). The treatment of first choice for SAHS remains continuous positive airway pressure (CPAP) therapy (6).

Dry mouth refers to a subjective sensation or oral dryness (xerostomia) or to a genuine decrease in salivary flow (hyposialia) (7), and is a very common sensation in the general population, with a prevalence of 5-46% (8). Some diseases such as Sjögren syndrome or diabetes, as well as different drugs and local factors such as smoking or mouth breathing, may cause dry mouth (9).

In relation to sleep, xerostomia on waking up has been reported in patients with SAHS (10,11), and people with xerostomia have been described as having poorer quality sleep (12). Furthermore, dry mouth is described by many patients as a side effect of CPAP, and may adversely affect adherence to therapy (13).

The present study describes the characteristics of xerostomia in the population with SAHS and contrasts its prevalence versus that found in healthy subjects. In addition, it compares the frequency of xerostomia in SAHS patients with and without continuous CPAP.

## Material and Methods

- Study design

A prospective case-control study was made of patients seen in Dr. Peset University Hospital (Valencia, Spain). The study complied with the guidelines of the Declaration of Helsinki for medical research in humans, and was approved by the local Clinical Research Ethics Committee. Written informed consent was obtained from all participating subjects.

We included patients between 25-75 years of age, with no relevant medical antecedents other than the disorders considered in the study, and who signed the corresponding informed consent document. Patients with an ASA score of IV or higher were excluded, as were pregnant women and patients receiving pharmacological treatment other than oral antidiabetic drugs in the last three months.

- SAHS group

The group of cases (with SAHS) consisted of patients from the Ear, Nose and Throat Department and Pneumology Department with a recent (< 12 months) diagnosis of SAHS based on the polysomnography (PSG) or respiratory polygraphy (RP) findings, according to the criteria of the American Academy of Sleep Medicine (AASM): AHI > 5/h plus associated symptoms, or AHI > 15/h. The AHI moreover evaluates the severity of SAHS, classifying it as mild (AHI: 5-14), moderate (AHI: 15-29) or severe (AHI ≥ 30). Within this group there was a subgroup of patients (70%) that had already started treatment with CPAP at the time of the exploration.

- Control group

The control group (without SAHS) consisted of healthy individuals acting as accompanying persons of patients visiting the Odontology Department. It included a subgroup of individuals (53%) in which the presence of SAHS was discarded based on objective parameters (respiratory polygraphy: AHI < 5/h) and another subgroup of individuals (47%) in which SAHS was discarded from the anamnesis (no snoring or daytime sleepiness as evidenced by a score of < 10 on the Epworth scale). Likewise, there were three subjects, initially included as controls, in which control respiratory polygraphic evaluation yielded AHI > 15/h.

- Anamnesis

Demographic data of all the participants were compiled, along with the general medical history (current and previous diseases, allergies, adverse drug reactions, regular medication and toxic habits: smoking and alcohol consumption) and body mass index (BMI). With regard to the frequency of xerostomia, all the participants completed a questionnaire scoring the frequency of the symptom on waking up and during the day, on a scale of 1-4 (1 = “never”, 2 = “occasionally”, 3 = “quite often” and “4 = “always”). A score of ≥ 3 was taken to indicate the presence of xerostomia.

- Statistical analysis

A descriptive analysis of the study variables was carried out, with calculation of the mean and standard deviation (SD), minimum and maximum, and median for continuous variables, and of absolute and relative frequencies (percentages) for categorical variables. Analysis of the homogeneity of groups was made with the Student t-test for independent samples (age and BMI for continuous variables) and the chi-squared test (for categorical variables). Simple binary logistic regression analysis was used to assess the probability of xerostomia, calculating the corresponding unadjusted (crude) odds ratio (OR) and 95% confidence interval (95%CI). Multiple regression analysis was used to adjust for independent variables (age and BMI). The level of statistical significance was established as 5% (α=0.05). The SPSS version 18.0 statistical package (IBM SPSS Statistics Inc., Chicago, IL, USA) was used throughout.

## Results

The total sample comprised 114 patients (60 SAHS cases and 54 non-SAHS controls), of which were 57 males and 57 females, with a mean age of 52.9 ± 10.2 years. In the SAHS group, the mean AHI score was 24.9. Twenty-five percent had mild SAHS, 40.0% moderate SAHS and 35.0% severe SAHS. On the other hand, 42 of the 60 SAHS patients (70%) used CPAP. Since the study was conducted in the public hospital setting, other therapeutic options, such as mandibular advancement devices (MADs), were not contemplated, since they are not financed by the Spanish national health system.

Table 1 shows some of the demographic and clinical characteristics of the study sample. The SAHS patients were comparatively older (SAHS: 55.4 ± 8.2 years; controls: 50.1 ± 11.5 years; *p*=0.007), and had a greater BMI (SAHS: 29.9 ± 4.1 kg/m2; controls: 23.9 ± 2.9 kg/m2; *p*<0.001). In contrast, both groups were homogeneous in terms of gender distribution (*p*=0.708), diabetes mellitus (*p*=0.056), smoking (*p*=0.256) and alcohol consumption (*p*=0.248).

**Table 1:**
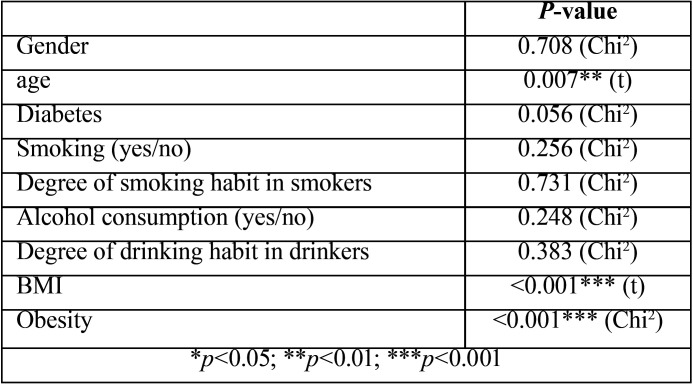
Homogeneity of the SAHS and control groups according to their demographic and clinical characteristics. Results of the chi-squared test (Chi2) and Student t-test (t) for independent samples.

Table 2 reports the frequency of dry mouth sensation after nighttime sleep and during the day. Taking a score of ≥ 2 as indicating the presence of xerostomia, the prevalence of dry mouth on waking up in the SAHS group was 45% (95%CI: 32.4-57.6) (mild SAHS: 20.0%; moderate SAHS: 62.5%; severe SAHS: 42.9%), versus 20.4% among the controls (95%CI: 9.6-31.1). Twenty-five percent of the patients with SAHS claimed to “always” experience the symptom on waking up, versus 7.4% of the controls. During the rest of the day, the dry mouth sensation decreased in both the SAHS group (21.7%; 95%CI: 11.2-32.1) and among the controls (9.3%; 95%CI: 3.1-20.3).

**Table 2:**
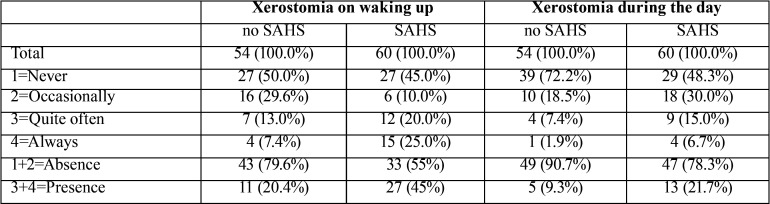
Frequency of xerostomia on waking up and during the day.

Initially, the patients with SAHS were estimated to have a 3.2-fold higher risk of xerostomia on waking up (*p*=0.006), and a 2.7-fold higher risk of experiencing xerostomia during the rest of the day (*p*=0.077). The association was therefore seen to be stronger when the symptom was evaluated after nighttime sleep. However, after adjusting for age and BMI, the analysis concluded that patient BMI was actually the factor explaining this association (*p*=0.007) (Table 3). We observed that for one same BMI, the difference in the probability of xerostomia between the SAHS group and the controls was inexistent (*p*=0.934). During the day, the influence of BMI was less apparent (*p*=0.097).

**Table 3:**
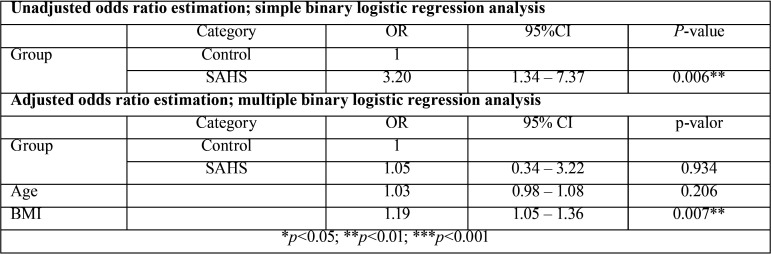
Correlation SAHS-xerostomia on waking up.

With regard to the relationship between the severity of SAHS and the presence of xerostomia on waking up, we found moderate SAHS to be associated to a greater frequency of xerostomia than mild SAHS (*p*=0.022). In the case of severe SAHS, the association was less manifest (*p*=0.171) (Table 4). After adjusting for age and BMI, and despite the strong influence of BMI (*p*=0.065) - indicating greater BMI to be correlated to an increased probability of xerostomia - the differences between moderate and mild SAHS persisted (*p*=0.022). No differences were noted during the day (*p*=0.956).

In relation to the use of CPAP, on comparing the frequency of xerostomia in the SAHS group between the patients with and without CPAP therapy, the prevalence was found to be 57.1% and 16.7%, respectively (*p*=0.008).

**Table 4:**
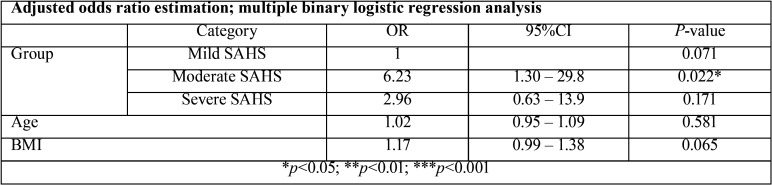
Correlation severity of SAHS-xerostomia on waking up.

## Discussion

The results of our study show that xerostomia, particularly on waking up in the morning, is a common symptom in patients with SAHS, and particularly in those receiving CPAP therapy. In 1985, Kales *et al.* (10) observed the presence of this symptom in 37 out of 50 patients with severe SAHS (74%).

In a later study of 668 patients scheduled for polysomnography, Oksenberg *et al.* (11) evaluated the presence of xerostomia in comparison with a series of 501 controls. The authors recorded xerostomia on waking up in 31.4% of the patients finally diagnosed with SAHS (22.4%, 34.5% and 40.7% in those with mild, moderate and severe SAHS, respectively), versus in 16.4% of the primary snorers and in 3.2% of the controls. The prevalence of this symptom increased with the severity of SAHS. The patients reporting dry mouth on waking up had a 2.33-fold higher risk of SAHS than the primary snorers, and a higher risk of suffering severe SAHS (OR= 4.03; 95%CI: 1.94-8.4).

In the same way as in the aforementioned study (11), we recorded a higher prevalence of xerostomia on waking up in the group of patients with SAHS: 45% (95%CI: 32.4-57.6) versus 20.4% in the control group (95%CI: 9.6-31.1). However, after adjusting for patient age and BMI, we found BMI - not the diagnosis of SAHS - to be the true factor responsible for the increased frequency of xerostomia (*p*=0.007). Although other authors have reported an increased incidence of salivary dysfunction in obese individuals (14,15), ours is the first study to identify BMI as the factor responsible for increased morning xerostomia among individuals with sleep apnea.

With regard to patient perception of the symptom during the day, the frequency of xerostomia in our sample was lower in both the SAHS group (21,7%) and among the controls (9,3%) than in either group on waking up in the morning (SAHS: 45%; controls: 20.4%). This seems logical, considering that salivary flow follows a circadian rhythm, with a marked drop during sleep (16). Moreover, in the SAHS group, the patients are largely mouth breathers (17), and therefore spend more time during nighttime sleep with the mouth open.

Previous studies in patients with SAHS have used scales to assess the frequency of a subjective symptom such as xerostomia (11,18). In our case we used the scale of Kreivi *et al.* (18), which scores the frequency of the symptom from 1-4, where 1 = “never”, 2 = “occasionally”, 3 = “quite often” and “4 = “always”. Likewise, we considered a patient score of 3-4 to indicate the presence of xerostomia. Oksenberg *et al.* (11) in turn used a similar scale with 5 degrees of frequency (“never”, “rarely”, “sometimes”, “often” or “almost always”). In contrast to our own study and that of Kreivi *et al.* (18), the mentioned authors only considered xerostomia to be present when the patient answered “almost always”. Possibly because of this stricter diagnostic criterion, the frequency of xerostomia on waking up in our study (45%) was greater than in that of Oksenberg *et al.* (31.4%).

On the other hand, López-Jornet *et al.* (12), in a sample of individuals previously diagnosed with hyposialia (unstimulated salivary flow < 0.2 ml/min), assessed different self-administered questionnaires, including one instrument related to sleep quality (Pittsburgh Sleep Quality Index) and another addressing daytime sleepiness (Epworth Sleepiness Scale). Dry mouth was found to be significantly associated to poorer sleep quality (*p*=0.006) and greater daytime sleepiness (*p*=0.010).

With regard to the influence of CPAP, in our study the patients with SAHS that used this therapy presented a higher prevalence of xerostomia on waking up (57.1%) in comparison with the SAHS patients that did not use CPAP (16.7%). The positive airway pressure of CPAP causes drying of the exposed mucosal tissues, and the oral mucosa is no exception in this regard. This would explain the increased prevalence of dry mouth in CPAP users. In addition, a high incidence of oral symptoms has been reported in association to CPAP, particularly in diabetic patients (19). In the study published by Ulander *et al.* (13), dry mouth was the side effect most often associated to CPAP therapy. Nevertheless, other authors suggest that dry mouth or throat could be improved with the use of CPAP (18,20). Regarding whether or not the use of a humidifier with CPAP could improve these symptoms, the data found in the literature are contradictory (18,21).

Based on the results obtained in our study, we can conclude that xerostomia is more frequent in individuals with sleep apnea than in healthy subjects - particularly when the symptom is assessed on waking up in the morning - and also in patients receiving CPAP therapy. In patients with SAHS, we found BMI to be significantly associated to an increased frequency of xerostomia. Likewise, xerostomia on waking up was more common in patients with moderate to severe SAHS than in patients with mild SAHS.

## References

[1] Phillipson EA (1993). Sleep apnea. A major public health problem. N Engl J Med.

[2] Dempsey JA, Veasey SC, Morgan BJ, O'Donnell CP (2010). Pathophysiology of sleep apnea. Physiol Rev.

[3] Peppard PE, Young T, Barnet JH, Palta M, Hagen EW, Hla KM (2013). Increased prevalence of sleep- disordered breathing in adults. Am J Epidemiol.

[4] Young T, Skatrud J, Peppard PE (2004). Risk factors for obstructive sleep apnea in adults. JAMA.

[5] Kushida CA, Littner MR, Morgenthaler T, Alessi CA, Bailey D, Coleman Jr J (2005). Practice parameters for the indications for polysomnography and related procedures: an update for 2005. Sleep.

[6] The Report of an American Academy of Sleep Medicine Task Force (1999). Sleep-related breathing disorders in adults: recommendations for syndrome definition and measurement techniques in clinical research. Sleep.

[7] Nederfors T (2000). Xerostomia and hyposalivation. Adv Dent Res.

[8] Villa A, Connell CL, Abati S (2014). Diagnosis and management of xerostomia and hyposalivation. Ther Clin Risk Manag.

[9] Millsop JW, Wang EA, Fazel N (2017). Etiology, evaluation, and management of xerostomia. Clin Dermatol.

[10] Kales A, Cadieux RJ, Bixler EO, Soldatos CR, Vela-Bueno A, Misoul CA (1985). Severe obstructive sleep apnea--I: Onset, clinical course, and characteristics. J Chronic Dis.

[11] Oksenberg A, Froom P, Melamed S (2006). Dry mouth upon awakening in obstructive sleep apnea. J Sleep Res.

[12] Lopez-Jornet P, Lucero Berdugo M, Fernandez-Pujante A, Castillo Felipe C, Zamora Lavella C, Pons-Fuster A (2016). Sleep quality in patients with xerostomia: a prospective and randomized case-control study. Acta Odontol Scand.

[13] Ulander M, Johansson MS, Ewaldh AE, Svanborg E, Broström A (2014). Side effects to continuous positive airway pressure treatment for obstructive sleep apnoea: changes over time and association to adherence. Sleep Breath.

[14] Flink H, Bergdahl M, Tegelberg A, Rosenblad A, Lagerlöf F (2008). Prevalence of hyposalivation in relation to general health, body mass index and remaining teeth in different age groups of adults. Community Dent Oral Epidemiol.

[15] Mathus-Vliegen EM, Nikkel D, Brand HS (2007). Oral aspects of obesity. Int Dent J.

[16] Thie NM, Kato T, Bader G, Montplaisir JY, Lavigne GJ (2002). The significance of saliva during sleep and the relevance of oromotor movements. Sleep Med Rev.

[17] Koutsourelakis I, Vagiakis E, Roussos C, Zakynthinos S (2006). Obstructive sleep apnoea and oral breathing in patients free of nasal obstruction. Eur Respir J.

[18] Kreivi HR, Virkkula P, Lehto J, Brander P (2010). Frequency of upper airway symptoms before and during continuous positive airway pressure treatment in patients with obstructive sleep apnea syndrome. Respiration.

[19] Tsuda H, Moritsuchi Y, Higuchi Y, Tsuda T (2016). Oral health under use of continuous positive airway pressure and interest in alternative therapy in patients with obstructive sleep apnoea: a questionnaire-based survey. Gerodontology.

[20] Avlonitou E, Kapsimalis F, Varouchakis G, Vardavas CI, Behrakis P (2012). Adherence to CPAP therapy improves quality of life and reduces symptoms among obstructive sleep apnea syndrome patients. Sleep Breath.

[21] Ruhle KH, Franke KJ, Domanski U, Nilius G (2011). Quality of life, compliance, sleep and nasopharyngeal side effects during CPAP therapy with and without controlled heated humidification. Sleep Breath.

